# Book Reviews

**Published:** 1911-11

**Authors:** 


### BOOK REVIEWS.

PATHOLOGICAL TECHNIC. Including Directions for the Performance of Autopsies and for Clinical Diagnosis by Laboratory Methods. By F. B. Mallory, M. D., Associate Professor of Pathology, Harvard Medical School; and J. H. Wright, M. D., Director of the Pathological Laboratory, Massachusetts General Hospital, Fifth Revised Edition. Octavo of 507 pages. Illustrated. Philadelphia and London: W. B. Saunders Company, 1911. Cloth, $3.00.

This book is an extremely valuable one to the general laboratory as well as to the busy practitioner; it takes up the subject of general laboratory work, as well as the mere simple tests used daily in the physician's laboratory.

Many methods used for tissue and blood analyses are also presented.

While concise it gives all the things sought for and is so classified that any one of the tests may be readily found and the method of making them simply and correctly given.

---

A TEXT-BOOK OF THE PRACTICE OF MEDICINE. By James M. Anders, M. D., Ph. D., LL. D., Professor of the Theory and Practice of Medicine and of Clinical Medicine, Medico-Chirurgical College, Philadelphia. Tenth Revised Edition. Octavo of 1328 pages, fully illustrated. Philadelphia and London: W. B. Saunders Company, 1911. Cloth, $5.50 net; half morocco, $7.00 net.

Anders does not need a formal introduction as his book is now in the 10th edition and those who have kept up with the progress in medicine on practice know that his work is one of the best.

Each subject is gone into thoroughly and, from the smallest signs and symptoms to the cardinal ones, are taken up in detail and are dealt with in a clear, scientific manner.

One point of great value is the differential diagnosis. This is important to the beginner who has not had the bedside training and who will have difficulty in differentiating the various signs and symptoms in disease.

---

CASE HISTORIES IN PEDIATRICS. A collection of Histories of Actual Patients, selected to illustrate the Diagnosis, Prognosis, and Treatment of the most important Diseases of Infancy and Childhood. Containing 320 Octavo pages and a Few Illustrations. Price, Express Prepaid, $3.00. Wm. Leonard, Publisher, 101 Tremont St., Boston.

Dr. Morse's book, Case Histories in Pediatrics, comprises one hundred actual histories selected to present as thoroughly as possible, for physicians, a Post-Graduate Clinical Course in Diseases of Children.

Each history is presented under the captions, History, Physical Examination, Diagnosis, Prognosis and Treatment.

Much attention is given to Diagnosis, which is thoroughly discussed by naming diagnoses which might with some reason have been made, and eliminating each in turn by careful reference to clinical signs and symptoms, the final diagnosis being that which cannot be set aside and which all symptoms support.

Treatment has received most attention. Both diagnosis and treatment have in each case been verified by subsequent history and are hence without error.

The book is original and thorough, and the most interesting as well as the most valuable in this department for the general practitioner.

---

A MANUAL OF MATERIA MEDICA. By E. Quin Thornton, M. D., Assistant Professor in Materia Medica in the Jefferson Medical College, Philadelphia. Cloth, $3.50 net. Lea & Febiger, Philadelphia and New York, 1911.

Professor Thornton has prepared a very thorough yet compact text-book and work of reference on the basis of therapeutics, namely the Materia Medica, a department which is coming in for renewed recognition as to its rightful importance. He opens with a section on posology, prescription writing, including its Latin essentials, incompatibles, weights and measures, and then proceeds to the body of the book, which is devoted to the description of all drugs, chemicals and preparations official in the U. S. Pharmacopoeia. In the third and closing part he gives a complete list of these preparations, rearranged according to pharmaceutical classes, with their composition and methods of preparation, thereby affording a laboratory guide. The author's long experience in teaching has given him definite views as to the field to be covered in a work of this kind, and also as to the best method of systematizing its presentation. The subject is one in which accuracy is peculiarly necessary, a characteristic well exemplified in the work in hand.

A MANUAL OF PRACTICE OF MEDICINE. By A. A. Stevens, A. M., M. D., Professor of Therapeutics and Clinical Medicine in the Woman's Medical College of Pennsylvania. Ninth Edition. Revised. 12mo of 573 ages, illustrated. Philadelphia and London: W. B. Saunders Company, 1911. Flexible leather, $2.50 net.

While concise, but to the point clear, no great part is devoted to differential diagnosis, while yet the manner in which each disease is taken up puts such distinct character is an unmistakable difference between the similar disease which makes a valuable differential point.

While no great number of rules are given these which are studied are good and each ingredient is given with a definite and distinct aim, and on the whole a brief compact little book which is well worth its space either on the shelves of lbiraries or the room it may take on its side of the busy physician's desk.

---

A MANUAL OF CLINICAL DIAGNOSIS BY MEANS OF LABORATORY METHODS. For Students, Hospital Physicians and Practitioners. By Charles E. Simon, M. D., Professor of Clinical Pathology and Experimental Medicine in the College of Physicians and Surgeons, Baltimore. Seventh edition, enlarged and thoroughly revised. Octavo, 780 pages, with 168 engravings and 25 plates. Cloth, $5.00 net. Lea & Febiger, Philadelphia and New York. 1911.

This splendid work needs no introduction to the profession. The cordial reception extended the six previous editions is proof of its value and usefulness.

---

ANATOMY. A Manual for Medical Students and Practitioners. By John F. Little, M. D., Asst. Demonstrator of Anatomy, Jefferson Medical College, Philadelphia. Second edition. Revised and enlarged. 490 pages. Illustrated with 75 engravings. Published by Lea & Febiger, Philadelphia.

This work presents more than the mere essentials of anatomy. To accomplish in a clear and concise manner in the limited space the less important points have yielded space to those of more practical bearing.

---

ONE HUNDRED SURGICAL PROBLEMS. The Experiences of Daily Practice Dissected and Explained. By James G. Mumford, M. D., Visiting Surgeon, Mass. General Hospital; Instructor in Surgery, Harvard Medical School; Fellow of the American Surgical Association, etc. Cloth, 354 pages. W. M. Leonard, Boston.

Mumford, the well-know surgeon, presents 100 case histories in a systematic manner and thus covers in a most instructive way the important phases of our common surgical problems. The book is well worth reading.

---

DISEASES OF THE STOMACH. With special reference to Treatment. By Chas. D. Aaron, Sc.D., M. D., Prof. of Gastrology, Detroit College of Medicine. With 42 illustrations and 21 plates. Published by Lea & Febiger, Philadelphia, Pa.

In this book the writer has covered the medical aspects of gastric disorders in such a manner as to answer the actual need of the practitioner. It is practical and therapeutic, and reflects the latest progress in gastrology.

